# The Immediate Economic Impact of Maternal Deaths on Rural Chinese Households

**DOI:** 10.1371/journal.pone.0038467

**Published:** 2012-06-06

**Authors:** Fang Ye, Haijun Wang, Dale Huntington, Hong Zhou, Yan Li, Fengzhi You, Jinhua Li, Wenlong Cui, Meiling Yao, Yan Wang, the study team for Economic Impact of Maternal Deaths in China

**Affiliations:** 1 Division of Maternal and Child Health, School of Public Health, Peking University Beijing, Beijing, China; 2 Department of Reproductive Health and Research, World Health Organization, Geneva, Switzerland; 3 School of Public Health, Kunming Medical College, Kunming, Yunnan, China; 4 Women’s Healthcare Section, Zhengzhou University 3rd Affiliated Hospital Zhengzhou, Henan, China; 5 Hebei Women and Children Health Center, Shijiazhuang, Hebei, China; Tehran University of Medical Sciences, Islamic Republic of Iran

## Abstract

**Objective:**

To identify the immediate economic impact of maternal death on rural Chinese households.

**Methods:**

Results are reported from a study that matched 195 households who had suffered a maternal death to 384 households that experienced a childbirth without maternal death in rural areas of three provinces in China, using quantitative questionnaire to compare differences of direct and indirect costs between two groups.

**Findings:**

The direct costs of a maternal death were significantly higher than the costs of a childbirth without a maternal death (US$4,119 vs. $370, *p<0.001*). More than 40% of the direct costs were attributed to funeral expenses. Hospitalization and emergency care expenses were the largest proportion of non-funeral direct costs and were higher in households with maternal death than the comparison group (US$2,248 vs. $305, *p<0.001*). To cover most of the high direct costs, 44.1% of affected households utilized compensation from hospitals, and the rest affected households (55.9%) utilized borrowing money or taking loans as major source of money to offset direct costs. The median economic burden of the direct (and non-reimbursed) costs of a maternal death was quite high - 37.0% of the household’s annual income, which was approximately 4 times as high as the threshold for an expense being considered catastrophic.

**Conclusion:**

The immediate direct costs of maternal deaths are extremely catastrophic for the rural Chinese households in three provinces studied.

## Introduction

The priority assigned to reducing the approximately 358,000 global maternal deaths per year is arguably at its highest levels since the 1994 International Conference on Population and Development and the establishment of the Millennium Development Goals (MDGs) [1], [2]. China bears a large burden of worldwide maternal mortality as the biggest developing country with 630 million women. Due to the large number of maternal deaths, China has been categorized into the 68 countdown countries to achieve MDG5 [3], [4].

The imperative to reduce risk due to childbirth is a fundamental human right, yet the economic aspects of maternal mortality are important to understand. However, that topic has not been widely explored in the literature on maternal health [5]. A systematic search on electronic databases of Pubmed, Embase, PAHO and Popline, and on home pages of major international organizations for published literature on the economic impact of maternal deaths did not identify any study on the costs of maternal death at household level. In recent years, severe acute maternal morbidity cases have been found to be a useful complement to investigation of maternal mortality [6], [7], and a few studies have estimated the costs to the health system of these cases but not maternal death [8]–[11].

There is much debate about whether maternal deaths are a symptom of poverty or whether they can push families into poverty. There is evidence on the co-existence of high maternal mortality ratios (MMR) and low level of economic development [12]. This is the case in China where MMR is substantially higher in provinces with lower GDP per capita than in wealthier provinces [13]. However, there is insufficient evidence on the economic costs of a maternal death, the strategies that poor families use to pay these expenses and the impact (immediate and lasting) on household economy. Therefore, we conducted this empirical study to identify both direct and indirect costs of maternal death, and the economic burden on households.

## Methods

The study matched eligible households experiencing maternal death with those of childbirth without maternal death (using selection criteria described below) to compare differences in indicators of direct and indirect costs as collected by direct interview within one to three months after maternal death or childbirth, (i.e., interviewers waited for 3 weeks after the maternal death before contacting the family). The results in this paper therefore describe the immediate economic shock of the maternal death. The study protocol and data collection instruments were approved by the ethical reviews boards of Peking University, and the World Health Organization. Signed informed consent statements were obtained prior to any interview. All interviews were entirely voluntary (no incentives were provided) and were timed to occur after the funeral period was completed.

### Study Setting

Three provinces (Hebei, Henan, and Yunnan) were purposively selected to represent settings with low, moderate, and high MMRs, with consideration given to comparable geographic area, population size, and transportation routes. The 2009 rural institutional delivery rate in Hebei, Henan, and Yunnan are all quite high (98.6%, 96.6%, and 83.8%, respectively) [14]. The rural areas in each province have experienced declines in MMR since 2005, and in 2009 the ratios were 13.0 (Hebei), 16.1 (Henan), and 38.2 (Yunnan) per 100,000 live births [14]–[18]. People’s living standards have increased in each of the provinces overall with wide disparities in per capita income between urban and rural areas in each province [19]–[24].

### Study Population

Households with maternal death (the affected group) were each matched to two households of childbirth without maternal death (the comparison group) according to the inclusion criteria as follows.

### Inclusion Criteria for the Households with Maternal Death

- Having maternal death within 3 months of the interview; Maternal death defined as the death of a woman while pregnant (≥28 weeks gestational age) or within 42 days of termination of pregnancy, not for accidental causes unrelated to the pregnancy.

### Matching Criteria for the Households of Childbirth without Maternal Death

- Having childbirth within 3 months of the interview, and;
- Living in the same administrative village, and;
- Similar economic status evaluated by the administrative village cadres (i.e. rich, moderate, poor) and;
- The household type is the same as that of the affected family before maternal death (i.e. nuclear or extended; with or without older children).

There were a total of 530 maternal deaths in Hebei, Henan, and Yunnan provinces between June 2009 and October 2010 (the study period). Of these cases, 40.9% (217 out of 530) were determined to be ineligible/did not meet the inclusion criteria and an additional 22.3% of the cases (118 out of 530) were eligible but refused to be interviewed. The principle reasons for refusing to be interviewed were unresolved medical malpractice legal suits, or extreme grief among the surviving family members. Overall, 36.8% of the total number of maternal deaths (195 out of 530) were eligible and accepted the interview. We compared the age and gestational week of maternal deaths between those accepted and refused to be interviewed. Results showed that no significant difference was observed (data not shown).

We purposively selected 384 matching households of childbirth without maternal death (using the selection criteria). There was no refusal from these families, i.e., 100% of the contacted families consented to take part in the study (the larger sample size for the comparison group was used to increase the statistical power of the analysis). The comparison households were interviewed within two days of the affected family’s intake interview. It’s noted that in Yunnan province six of the affected households were matched with only one comparison due to difficulties in identifying a second eligible comparison family in the remote and under-populated areas.

### Data Collection

Potentially eligible families were identified by the County Maternal and Child Health Office in the selected provinces. Preliminary contact was made by one member of the provincial study team within three months (but not under three weeks) of the maternal death or childbirth. After eligibility was confirmed and informed consent was made, the person in charge of the household economy was invited to a face-to-face interview. Each interview was conducted in Chinese or local dialect by members of the study team who had received standardized training on interviewing techniques. Additional training was provided to interviewers by a psychological expert with experience in bereavement, in order to further reduce the potential harm to the study’s subjects. During each interview, we collected data with quantitative questionnaires, which was designed after a literature review of cost-of-illness studies [25], [26], expert consultation and piloting study in counties with social-economic characteristics similar to the study sites.

The questionnaire included questions on the household demographic status (age, household size, whether the mother was minority (In China, “minority” means non-Han ethnicity, including Hui, Yi, Hani, etc.), whether the family was nuclear family, etc.), household economic status (income, expenditure, etc.), direct and indirect costs of maternal death or childbirth, and coping strategy. Direct costs, i.e. out-of-pocket expenditures, included hospitalization and emergency costs, transportation costs, extra costs, and funeral costs, those were being listed as four items in the questionnaire. To estimate indirect costs, we collected self-reported lost work-day during the hospitalization and medical treatment, funeral, and prolonged bereavement, then the total lost work-days were multiplied by estimated daily income per capita(derived from household annual income divided by household size and by 365). Coping strategy, defined as sources of money used to offset direct costs, were also obtained, which included positive sources(money supported by external parties, such as hospital compensation, gifts from relatives or friends, medical insurance reimbursement, government cash assistance) and negative sources (money from household by selling assets, mobilizing available cash or savings, borrowing money or taking loans).

### Data Analyses

The database was developed using the Epidata 3.1 software. Statistical analyses were conducted with SPSS Version 10.0. The demographic characteristics (age, household size, whether the mother was minority, whether the family was nuclear family etc.), direct and indirect costs, and coping strategies were compared between the affected and comparison group. For categorical variables and proportion comparison, χ^2^ or Fisher’s exact tests were used for comparison. For continuous variables, difference in means for normally distributed data was tested by t-test, and other continuous variable, such as household annual expenditure, time and costs data which had positively skewed distributions, were tested for differences by rank-sum test. *P*<0.05 was considered to be statistically significant. The exchange rate (US$1 =  ¥6.7) was used to convert the payment from Chinese RMB (¥) to US$.

## Results

### Sample Characteristics

The sample’s demographic characteristics reveal a successful matching of the two study groups (Table 1). The two groups did not differ in household size (*p = 0.803*), proportion of nuclear families (*p = 0.846*), proportion of minorities (*p = 0.290*), and annual household expenditure(*p = 0.117*). The women in affected group tended to be older than those in comparison group (*p<0.001*). Although women in the affected group were more likely to have delivered at home than in the comparison group (13.6% vs. 4.4%, *p<0.001*), there are approximately 65% of women in both study groups delivered in county or upper-level hospitals. A higher proportion of women in the affected group had a cesarean section than in the comparison group (48.3% vs. 31.8%, *p<0.001*).

**Table 1:** Sample characteristics by province and study group.

Demographic Characteristics	Hebei		Henan		Yunnan		Total		
	Affected(n = 38)	Comparison(n = 76)	Affected(n = 58)	Comparison(n = 116)	Affected(n = 99)	Comparison(n = 192)	Affected(n = 195)	Comparison(n = 384)	*P- value*
Median age of the woman(Range in Years)	30.7 (20.7–41.0)	28.2 (19.8–40.4)	30.2 (18.3–45.2)	25.5 (16.2–42.5)	28.4 (18.3–44.7)	25.5 (14.8–42.5)	29.4 (18.3–45.2)	26.0 (14.8–42.5)	<0.001^*^
Household size (Mean ± SD)	4.3±1.5	4.2±1.6	4.6±1.2	4.8±1.5	4.5±1.5	4.5±1.7	4.5±1.	4.5±1.6	*0.803^#^*
% of nuclear families	42.1	43.4	27.6	21.6	31.3	32.8	32.3	31.5	*0.846^†^*
% of minorities*^‡^*	0.0	2.6	1.7	0.9	52.5	44.8	27.2	23.2	*0.290^†^*
Household annual expenditure (US$)(Mean ± SD)	6480.9±9612.5	5677.7±6402.2	3621.1±3414.4	3675.6±3708.0	4048.2±6081.0	4691.5±3882.5	4395.3±6389.1	4579.8±4490.5	0.117^*^

Affected: households with a maternal death; Comparison: matched households without maternal death. The p-values presented are for the comparison of Total Affected versus Total Comparison. SD: Standard Deviation. *^‡^*In China, “minority” means non-Han ethnicity, including Hui, Yi, Hani, etc. ^*^ Rank-sum test. ^#^ t-test. *^†^* Chi-square test.

### Direct Costs

**Figure 1:**
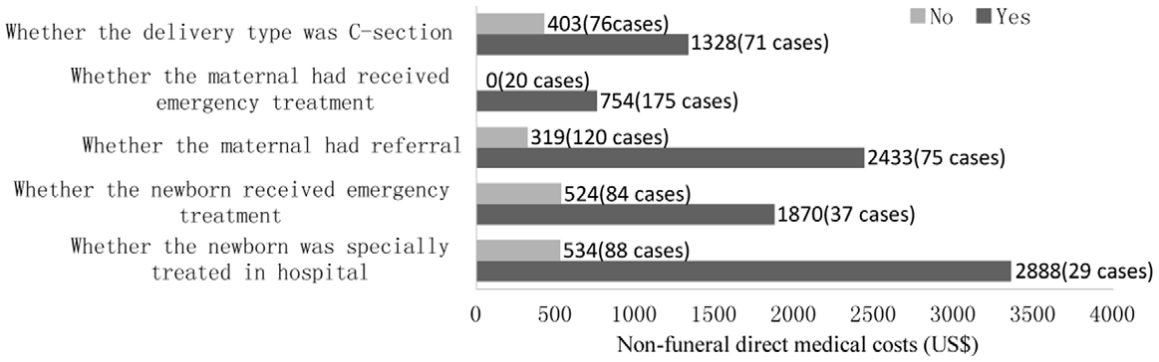
Non-funeral direct costs for households with maternal death. Note: costs presented here are sub-group analysis based on the affected group (n = 195); 48 cases were missing in category of delivery type (48 women in affected group died before childbirth and no delivery type was concerned); 74 cases were missing in category of whether the newborn received emergency treatment(48 women in affected group died before childbirth, 24 cases were stillbirths, and 2 cases were unknown); 78 cases were missing in category of whether the newborn was specially treated in hospital (48 women in affected group died before childbirth, 24 cases were stillbirths, and 6 cases were unknown).

Direct costs were assessed as the household’s out-of-pocket expenditures associated with maternal death or childbirth (Table 2). The total direct costs with maternal death were more than ten times higher than those without maternal death (US$4,119 vs. $370, *p<0.001*). Funeral costs made up the largest category of the direct costs for the affected group (US$1,654). The non-funeral direct costs were quite substantial in affected group (US$2,464) and were more than six times higher than in comparison group (US$370)(*p<0.001*), reflecting the difference in hospitalization and emergency costs (US$2,248/case of maternal death versus US$305/case of childbirth without maternal death). Excluding 12 outliers (8 in affected group and 4 in unaffected group) higher than 3 SDs of mean, direct costs were still significantly higher in affected group compared with unaffected group (mean $3446 vs. $312, median $2452 vs. $224, *p<0.001*). We conducted multi-variable linear regression to control for possible confounders, including province, age of the mother, whether the mother was literate, whether the mother had pregnancy complications etc. The results showed that after controlling these characteristics, the non-funeral direct costs were still significantly higher in affected group, and the ratio between two groups increased slightly (3.32 vs. 3.46) (see Table S1). The non-funeral direct costs were principally for intensive care for the maternal and newborn, and referral expenses (Figure 1).

**Table 2:** Direct costs (all types) by study group (US$).

Type of Direct Costs*	Affected (n = 195)					Comparison(n = 384)					
	Mean(% of total direct costs)	Median	IQR	Min	Max	Mean(% of total direct costs)	Median	IQR	Min	Max	*P-value^‡^*
**Direct medical cost**											
Hospitalization and emergency costs	2248(54.6%)	463	119–2388	0	22582	305(82.4%)	149	97–352	0	7836	*<0.001*
**Direct non-medical cost**											
Transportation costs	86(2.1%)	15	0–94	0	1433	11(3.0%)	6	0–15	0	149	*<0.001*
Extra costs (accommodation, nutrition, etc.)	131(3.2%)	15	0–67	0	4478	54(14.6%)	30	7–74	0	896	*0.003*
Funeral costs	1654(40.2%)	1418	597–2239	0	10448	NA	NA	NA	NA	NA	*NA*
Total direct costs ^#^	4119(100.0%)	2612	1425–5067	0	26866	370(100.0%)	224	118–459	0	8433	*<0.001*
Non-funeral direct costs†	2464	594	179–2604	0	24627	370	224	118–459	0	8433	*<0.001*

Affected: households with a maternal death; Comparison: matched households without maternal death. N/A: Not Applicable.

* Exchange rate (US$1 =  ¥6.7). # Total direct costs are the sum of hospitalization and emergency costs, transportation costs, extra costs and funeral costs.

† Non-funeral direct costs are the sum of hospitalization and emergency costs, transportation costs, and extra costs. ‡ Rank-sum test.

### Indirect Costs

Indirect costs included lost household economic productivity during the hospitalization and medical treatment, funeral, and prolonged bereavement (Table 3). Families who experienced a childbirth without maternal death were more likely to report taking a longer period away from working than families with a maternal death during the hospitalization(6 work-days vs. 4 work-days, *p = 0.004*). However the families with a maternal death experienced a substantial number of lost working days during the funeral and bereavement period. Results from self-reported lost work-days were summed and multiplied by the estimated daily income per capita (derived from household annual income divided by household size and by 365) to estimate total lost economic productivity. The total lost economic productivity of households with maternal death was significantly higher than that in comparison group (US$154 vs. $15, *p<0.001*). Excluding 9 outliers (3 in affected group and 6 in unaffected group) higher than 3 SDs of mean, indirect costs were still significantly higher in affected group compared with unaffected group (mean $130 vs. $14, median $96 vs. $10, *p<0.001*).

**Table 3:** Indirect costs by study group.

Type of Indirect cost	Affected (n = 195)					Comparison (n = 384)					
	Mean(% of total lost work-days)	Median	IQR	Min	Max	Mean(% of total lost work-days)	Median	IQR	Min	Max	*P-value^ #^*
Lost number of person work-days for taking care ofthe women during hospitalization/childbirth	12(12.9)	4	2–13	0	120	9(100.0)	6	*3–14*	0	114	*0.004*
Lost person work-days of family members during funeral period	35(37.6)	12	4–27	0	1245	N/A	N/A	N/A	N/A	N/A	*N/A*
Lost person work-day of husband post-funeral period due to bereavement	46(49.5)	45	20–64	0	121	N/A	N/A	N/A	N/A	N/A	*N/A*
**Total number of lost person work-days**	93(100.0)	74	46–103	3	1355	9(100.0)	6	*3–14*	0	114	*<0.001*
Total lost economic productivity (US$)*	154	98	57–156	3	2286	15	10	*4–21*	0	134	*<0.001*

Affected: households with a maternal death; Comparison: matched households without maternal death. N/A: Not Applicable * Total lost economic productivity equals total number of lost person work-days multiply estimated daily income per capita(derived from household annual income divided by household size and by 365); Exchange rate(US$1 =  ¥6.7). # Rank-sum test.

### Sources of Financial Support Used to Offset Direct Costs

Respondents identified several sources of income which were used to raise funds paid out as direct costs of the maternal death or childbirth without maternal death (Table 4). These different sources were either positive (money supported by external parties, such as hospital compensation, gifts from relatives or friends, medical insurance reimbursement, government cash assistance) or negative (money from household by selling assets, mobilizing available cash or savings, borrowing money or taking loans).

Slightly less than one-half (44.1%) of the affected households received compensation from the hospital, and 29.2% of the affected households had all of the direct costs for the maternal death paid by the hospital where the woman died. Among the approximately one-half (55.9%) of the affected households that did not receive any payment from the hospital, their largest source of revenue for paying the direct costs of the maternal death came from borrowing money or taking loans (65.1% of the direct costs).

**Table 4:** Sources of financial support used to offset direct costs by two study groups.

	Affected group(n = 195)			Comparison group(n = 384)
	Sufficient compensation from external parties to cover alldirect costs (n = 57, 29.2%)	Insufficient compensation from external parties to cover alldirect costs (n = 29, 14.9%)	No compensation received from Hospital, only from othersources (n = 109, 55.9%)	
Mean of direct costs ($US)	3049	6149	4138	370
**Percentage of direct costs supported by external parties**				
Hospital compensation	100.0	47.0	0.0	0.0
Gifts from relatives or friends	0.0	4.8	8.2	32.9
Medical insurance reimbursement	0.0	4.3	5.8	24.2
Government cash assistance	0.0	0.1	0.5	0.0
**Percentage of direct costs paid by household**				
Selling assets	0.0	1.8	2.1	0.1
Mobilizing available cash or savings	0.0	13.3	18.3	24.8
Borrowing money or taking loans	0.0	28.7	65.1	18.0

Affected: households with a maternal death; Comparison: matched households without maternal death.

In contrast, the households of childbirth without maternal death utilized a narrower range of financial support mechanisms, with the majority reporting supports that came from gifts from relatives or friends (32.9%) or by drawing upon the household savings (24.8%).

### Economic Burden

The expenditure of a disease is considered to be catastrophic for the household economy if its economic burden crosses certain threshold, which has different calculation methods by different researchers. We employed two more frequently used methods in this study.

One method is direct costs expressed as a percentage of the household’s total annual income, and the threshold is 10.0% [27], [28]. We applied this formula and found that, the median economic burden of a maternal death was 37.0% of the household's annual income, compared to 0*·*0% for households without maternal death (the means were 101.2% vs.8.8%) (*p<0.001*). Without excluding the amounts reimbursed by hospitals, insurance, government or neighbors, the median of economic burden was equal to 128.8% of the household’s annual income, compared to 8.0% of the household’s annual income for childbirth without maternal death (the means were 244.4% vs.17.3%) (*p<0.001*).

An alternative method for determining catastrophic health expenditures defines the direct costs excluding reimbursed money higher than 40% of household expenditures after excluding food costs [29], [30]. Applying this method, we found the median burden of a maternal death was 56.2%, compared to 0.0% in comparison group(the means were 229.6% vs.23.7%) (*p<0.001*).

The economic burden of a maternal death observed in this study is much higher than that associated with other priority diseases. For example, cost-of-illness studies that used the 10% threshold definition on malaria reported a direct economic burden from 2.0% to 2.9% for malaria [31], and a series of tuberculosis (TB) studies demonstrated direct economic burden from 8.3% in Zambia to 13.0% in India [32], [33].

## Discussion

The findings that the direct medical costs of a maternal death (i.e., excluding funeral expenses and costs that were reimbursed by others) were significantly higher than the costs of childbirth without maternal death are not unexpected. However the high proportion of families who had catastrophic health expenditures as a result of a maternal death draws attention. The affected families were more vulnerable to catastrophic health expenditures than the comparison group, due to their lower levels of household income, and the overall magnitude of the economic shock is not trival and should be a focus of serious attention by local and provincial authorities.

The costs associated with a maternal death (direct and indirect) will clearly vary by setting and should not be overly generalized. For example, the direct non-funeral costs observed in our study are higher than the cost of treatment of life-threatening obstetric complications reported in Burkina Faso (US$48) [10], Benin (US$256), and Ghana (US$115) [9]. This can be attributed to differences in the intensity of medical services provided, level of economic development in the study’s setting, investigation year, and medical condition generating the costs (life-threatening obstetric complications vs. maternal death). However in each setting the relative economic burden is an important consideration for national policy concerned with economic development, and equitable financing schemes for health care and, as such, deserved to be examined such as what was done in China by this study.

Although not formal policy, the common practice of hospitals to provide cash payments to the families who suffered a maternal death does work to alleviate some of the immediate economic shock of the high medical care costs. Approximately one-third of the affected families reported that these payments covered all of their direct costs for the maternal death. The terms of the payments were not explored by this study, but credible anecdotal evidence indicates that it can be linked to an agreement not to pursue legal suit for malpractice against the hospital or physician. Often the payments were offered only after family members protested angrily in the hospital compounds. These payments are largely ad hoc with little record established on the condition and criteria for determining the amount or source of budget used for the payments. Given the frequency of these types of mal-practice payments encountered in our study, it seems reasonable to suggest that this is a priority area for government regulation. Additionally, little is known about the likelihood of a hospital to turn away complicated or near death maternal patients for fear of being liable to pay out compensation to the surviving family members. More research is needed on this troubling new trend in rural China.

The households with a maternal death who did not have all direct costs paid by the hospitals managed the expenses by borrowing money or taking loans, drawing down their savings and selling assets – thereby pushing poor households even deeper into poverty. Local government did provide some measure of financial relief, amounting to only 0.5% of direct costs of these households, but at too low a level to make any appreciable difference on the economic burden caused by paying for the end of life medical procedures surrounding the maternal death. In addition to regulating the informal payments made by hospitals in the case of maternal deaths, local governments need to reconsider the amounts made available to families who have experienced catastrophic health expenditures. Financial assistance such as new types of insurance for pregnant women, increased action from government and NGOs programs associated with financial aid to the poor, children schooling, or elderly pension are suggested as avenues for providing emergency financial relief.

Households with catastrophic economic burden coupled with fewer assets, reduced savings and increased debt are likely to suffer consequences that affect all the surviving family members, including reduced expenditures for food and education. Clearly the high economic burden that the maternal death causes has resulted in a severe economic shock in the short term. The resilience of the affected families to this shock over time is unknown and future research that tracks the longer term effects over time are needed to fully understand the implications of the immediate catastrophic health expenditures associated with a maternal death.

To our knowledge, the present study is the first to compare the direct costs, indirect costs and economic burden between the households which experienced childbirth with and without maternal death in rural areas of China, or elsewhere in the world. Results from our study are generalizable only within China, but can inform understanding of the multiple consequences of maternal death in other settings, and provide the first reference on the economic implications of this tragedy. The consequence of maternal mortality is extremely difficult to measure, because it is a relatively rare event. We had overcome this difficulty to achieve the relatively large sample by utilizing a national surveillance network of maternal health in China. The field work for gathering this evidence was quite laborious as extra effort was made to recruit subjects, as most fatalities occurred in under-populated areas with inconvenient transportation. We hope that our first study on this topic will encourage others to undertake similar research, and create evidence for successful advocacy to increase the financial support provided to poor families who have suffered a maternal death.

## Supporting Information

Table S1 Impact of possible confounders on non-funeral direct costs: multi-variable linear regression.
(DOC)
