# Supplementary material for: The Immediate Economic Impact of Maternal Deaths on Rural Chinese Households
Source: PLoS One. 2012 Jun 6;7(6):e38467. doi: 10.1371/journal.pone.0038467 (PMC3368847; doi:10.1371/journal.pone.0038467)
Supplement: Table S1 — Impact of possible confounders on non-funeral direct costs: multi-variable linear regression. (DOC) [file pone.0038467.s001.doc]

Table S1 Impact of possible confounders on non-funeral direct costs*: multi-variable linear regression

|  | Crude model | | | Adjusted for possible confounders | | |
| --- | --- | --- | --- | --- | --- | --- |
|  | β** | exp(β)*** | *P* | β** | exp(β)*** | *P* |
| **Whether the mother died** | 1.20 | 3.32 | *<0.01* | 1.24 | 3.46 | *<0.01* |
| **Province** |  |  |  |  |  |  |
| Hebei | - | - | - | 0.30 | 1.35 | *0.05* |
| Henan | - | - | - | Reference group | Reference group | *Reference group* |
| Yunnan | - | - | - | -0.26 | 0.77 | *0.06* |
| **Age of the mother** | - | - | - | -0.08 | 0.92 | *0.39* |
| **Whether the mother was literate** | - | - | - | 0.33 | 1.39 | *0.08* |
| **Whether the mother was Han ethnicity** | - | - | - | 0.19 | 1.21 | *0.20* |
| **Whether the mother had pregnancy complications** | - | - | - | 0.26 | 1.30 | *0.06* |

* Non-funeral direct costs had skewed distribution and were logarithmically transformed before linear regression.

**β represents the coefficient in the linear regression model.

***Exp(β) represents ratio of non-funeral direct costs between households with and without the factor.
